# Does tattoo exposure increase the risk of cutaneous melanoma? A population-based case-control study

**DOI:** 10.1007/s10654-025-01326-6

**Published:** 2025-11-24

**Authors:** Emelie Rietz Liljedahl, Kari Nielsen, Malin Engfeldt, Anna Saxne Jöud, Christel Nielsen

**Affiliations:** 1 Division of Occupational and Environmental Medicine, Department of Laboratory Medicine, Lund University, Lund, Sweden; 2 Department of Dermatology, Skåne University Hospital, Lund, Sweden; 3 Dermatology, Department of Clinical Sciences, Lund University, Lund, Sweden; 4 Department of Occupational and Environmental Medicine, Region Skåne, Lund, Sweden; 5 Division of Orthopaedics, Clinical Sciences Lund, Lund University and Skåne University Hospital, Lund, Sweden; 6 Clinical Pharmacology, Pharmacy and Environmental Medicine, Institute of Public Health, University of Southern Denmark, Odense, Denmark; 7 Lund University Occupational and Environmental Medicine, Medicon Village (402A), Scheelevägen 8, Lund, 223 81 Sweden

**Keywords:** Tattoo, Melanoma skin cancer, Risk factors, Lifestyle factors, Health risks

## Abstract

The incidence of cutaneous melanoma (CM) has risen sharply over the past 30 years, coinciding with the rapidly growing tattoo trend. In Sweden, 20% of the population is tattooed. Repeated reports of the presence of carcinogenic chemicals, such as polyaromatic hydrocarbons, aromatic amines and heavy metals in tattoo ink justifies the investigation of CM risk in relation to tattooing. We aimed to investigate the potential association between tattoo exposure and CM. We identified 2880 individuals who were diagnosed with CM at age 20–60 years, in the Swedish National Cancer Register. For each case, we sampled three random age- and sex-matched controls from the Swedish Total Population Register. Exposure data and data on potential confounders were collected through a questionnaire in 2021. We estimated the relative risk of CM in tattooed compared with nontattooed individuals using multivariable logistic regression, rendering incidence rate ratios (IRR). Of the participants, 22% of the cases (354/1598) had a tattoo before the index date, vs. 20% of the controls (815/4097). We observed an adjusted relative risk of CM in tattooed compared to nontattooed individuals of 1.29 (95% confidence interval [CI]: 1.07–1.56). The results suggested that tattoos may be a risk factor for CM, but further studies are needed to establish causality.

**Supplementary Information:**

The online version contains supplementary material available at 10.1007/s10654-025-01326-6.

## Introduction

The incidence of cutaneous melanoma has risen sharply over the past 30 years [1]. This has primarily been attributed to increased exposure to ultraviolet (UV) radiation [2], but chemical exposures are suspected to also play a role in the development of cutaneous melanoma, especially exposure to polyaromatic hydrocarbons (PAH) in occupational settings [3]. Risk factors related to lifestyle, such as tattoos, have been studied less. Historical tattoo prevalence data from Sweden are missing, but a Danish study of blood donors in 2001 showed that 10% (56/576) had a tattoo [4]. Recent estimates show that, in Sweden, 20% of the population is tattooed and the prevalence is above 30% in the population younger than 40 years [5]. The majority of people get their first tattoo at a young age – 18–35 years – and 20% are even younger [6]. This implies exposure to the inks for almost the entire life course, but little is known about the long-term health effects. Therefore, regulatory authorities have highlighted the need to clarify a potential link between tattoos and cancer.

Tattoo ink contains colour pigments – which are often the source of carcinogenic chemicals in tattoo ink – as well as solvents, preservatives, and impurities. Azo pigments dominate the organic colourants used in tattoo ink, which is problematic as they can be cleaved to carcinogenic aromatic amines, a process that is accelerated by UV radiation from exposure to sun, sunbeds or laser treatment [7]. Black ink often contains high amounts of PAH [8], some of which the International Agency for Research on Cancer [9] has classified as either carcinogenic or potentially carcinogenic, together with shading additives such as titanium dioxide [10]. Finally, heavy metals feature prominently irrespective of ink colour [11].

In January 2022, an EU regulation that imposes concentration limits for 4,000 chemicals in tattoo ink entered into force under Registration, Evaluation, Authorization and Restriction of Chemicals (REACH) [12]. Up until then, the market for tattoo ink had been relatively unregulated. Market controls by authorities show that inks contained toxic chemicals at levels above the stipulated limit values both before [13, 14] and after [15] the implementation of the EU regulation. The situation is often further complicated by fraudulent labelling of tattoo ink, where banned pigments are masked by listing legal pigments or label forging [13, 15].

An experimental study in mice has provided some evidence that red ink containing 2-anisidine may function as a weak cocarcinogen after irradiation with UV light [16]. However, no such studies have been undertaken to investigate the effect on CM onset.

Numerous case reports have described skin cancer in tattoos [17], but case reports suffer from inherent publication bias and cannot be used to infer causation. A recent Danish study of twins found an increased risk of skin cancer among tattooed individuals, but they only reported hazard ratios for melanoma and non-melanoma skin cancers combined [18]. Our aim was to answer if tattoo exposure increases the risk of cutaneous melanoma. Additionally, we hypothesized that exposure to UV radiation may act as an effect modifier, as it accelerates pigment breakdown into aromatic amines. We employed a population-based case-control design using national administrative registers and collected detailed exposure data using questionnaires.

## Methods

### Study design

In this population-based case-control study nested within the Swedish total population, all individuals with a diagnosis of nevus with severe atypia, cutaneous melanoma in situ or invasive cutaneous melanoma aged 20–60 years at diagnosis in 2017 (i.e. the index year) were identified in the National Cancer Register. Here, healthcare providers are obliged to report all primary neoplasms according to the Act on Health Data Registers (1998:543). The coverage of skin cancers is above 99% [19], and 99% of tumours in the skin are morphologically confirmed [20].

Three matched controls per case of the same age and sex were randomly sampled in the Total Population Register on the index date using incidence density sampling with person-time as the underlying timescale.

The study population was cross-referenced with the National Patient Register to exclude individuals at risk of suffering psychological distress due to study participation (i.e., F20-29 in the International Statistical Classification of Diseases and Related Health Problems [ICD], 10th Revision). If cases were deceased, the date of death was retrieved from the National Cause of Death Register. Next-of-kin were identified in the Total Population Register or the Multi-Generation Register and contacted to limit the impact of potential survivorship bias. Finally, indicator variables of socioeconomic status (SES) were retrieved from the Longitudinal Integration Database for Health Insurance and Labor Market Studies.

### Exposure assessment

The survey was administered by Statistics Sweden and conducted between February and April 2021. We used a structured questionnaire to collect exposure data; it has been described in detail elsewhere [5].

In brief, we defined tattoos as permanent motifs obtained for decorative, cosmetic (i.e., permanent or semi-permanent makeup and microblading), or medical (i.e., in reconstruction of skin after surgery) reasons. Respondents were explicitly asked to also consider removed tattoos.

The study was powered to assess exposure as a dichotomous variable, i.e., as presence or absence of at least one tattoo. We assessed the area of the tattooed body surface as predefined increments on a categorical scale (i.e., < 1 palm, 1–5 palms, >5 palms), and as the number of undergone tattoo sessions.

We collected data on the respondents’ age when they obtained their first tattoo, and tattoo characteristics including colours, anatomical location, tattooist (professional or nonprofessional), and in which geographic region(s) the tattoos had been received.

The questionnaire contained validated items on recreational and occupational sun exposure [21], history of sunburns and use of tanning beds. In addition, the questionnaire asked about skin characteristics, i.e., pigmentation according to the Fitzpatrick Skin Phototype Classification type I-VI [22], and reaction to sun exposure derived from Fitzpatrick Skin Phototype Classification type I-IV. Finally, it also covered smoking.

### Outcome

We defined incident primary cutaneous melanoma tumours according to ICD-O/3.2 (International Classification of Diseases for Oncology, 3rd Edition) typography code C44, in combination with any of the morphology codes listed in Table 1. In Sweden, dysplastic nevi (melanocytic nevi with severe atypia) are reported to the cancer registry. They are by definition not a cancerous lesion, but it is difficult to distinguish them clinically and often histopathologically from cutaneous melanoma in situ. In the present paper, we included them in the *in situ/*precursor lesion group of cutaneous melanomas because, in Sweden, that is how they are administered clinically. The main analysis of the present work refers to “overall cutaneous melanoma” and includes both precursor lesions and invasive cutaneous melanomas.

**Table 1:** International classification of diseases for Oncology, 3rd Edition, morphology codes (SNOMED; systematized nomenclature of Medicine) used in combination with typography code C44 to identify cases with cutaneous melanoma and registered associated diagnoses in the National cancer Register, with the number of cases (n) included in the present study

Morphology type	SNOMED	*n*
In situ/precursor lesions		
Melanocytic nevus with severe atypia	8727/0b	717
Lentigo maligna	8742/2b	65
Acral lentiginous melanoma, in situ	8744/2	4
Atypical Spitz’s nevus	8770/1b	9
Pigmented epithelioid melanocytoma/epithelioid blue nevus	8780/1b	–
Invasive cutaneous melanomas		
Superficial spreading melanoma	8743/3	627
Nevoid melanoma and melanoma not otherwise specified	8720/3	121
Lentigo malignant melanoma	8742/3	18
Acral lentiginous melanoma	8744/3	9
Nodular melanoma	8721/3	25
Desmoplastic melanoma	8745/3	–
Malignant melanoma in a giant pigmented nevus	8761/3	–
Amelanotic melanoma	8730/3	–
Mixed epithelioid and spindle cell melanoma	8770/3	3
Melanoma from blue nevus	8780/3	–

### Study size

We determined the sample size through an a priori statistical power analysis considering tattoo status as a dichotomous variable. We wanted to be able to detect an odds ratio of 1.3 with 80% power, assuming a tattoo prevalence of 17% among controls [23]. This would be achieved by including 1300 cases and 2 controls per case. Acknowledging that questionnaires often suffer from low response rates, we scaled up the study population to 3000 cases and 3 controls per case to ensure sufficient power.

### Statistical methods

For the questionnaire, an aggregated dropout analysis of characteristics of the participants and nonparticipants was performed by Statistics Sweden. Individual-level data from nonparticipants were not available.

To be considered exposed, participants were required to have obtained their first tattoo *before* the index year, to ascertain temporality between exposure and outcome.

For tattooed cases, we assessed the correlation between the tumour site and the anatomical location of tattoos using Spearman’s rho.

We used Directed Acyclic Graphs (DAG) in the design phase of the study to visualize potential confounders of the potential association between tattoo exposure and cutaneous melanoma (Figure S1). These assumptions informed the data collection strategy. We constructed an UV-exposure index to describe the cumulative UV exposure from numerous exposure sources according to the questionnaire items: “Occupational sun exposure (April-September),” “Recreational sun travel” and “Sunbed use.” Respondents were placed in the high category if they provided ≥ 1 response in the high exposure category on any of these items, in the medium category if they did not provide a response in any of the high categories and ≥ 1 response in a medium category, and in the low category if they only provided responses in the lowest categories.

We also created a phenotypic risk index to account for skin characteristics using the items skin pigmentation and skin reaction after sun exposure [22]. High-risk participants were those who reported “Skin reaction to first sun exposure”: “Burns (sometimes with blisters) and pain for at least 48 h” or “Burns with pain that disappears within 24 h,” regardless of their skin tone. Medium-risk participants were those who reported “Skin reaction to first sun exposure”: “First burn, then tan,” regardless of skin tone, and “Very fair” or “Fair” skin tone in combination with “Skin reaction to first sun exposure”: “Tan immediately.” Low-risk participants were those who reported “Medium,” “Olive,” “Brown” or “Dark brown” skin tone and reported “Skin reaction to first sun exposure”: “Tan immediately.”

We used logistic regression to investigate the association between tattoo exposure and cutaneous melanoma. The odds ratio offers an unbiased estimate of the incidence rate ratio (IRR) in the underlying population in case-control studies with incidence-density sampling. Exposure was modelled as a dichotomous variable: tattoo exposure (yes; no), as ordinal variables: size of tattoo (< 1 hand; >1 hand [1–5 hands and >5 hands were collapsed into the same category because overestimation of self-assessed tattoo size has been reported previously] [24]) and number of tattoo sessions (1; 2–3; ≥ 4 tattoo sessions), and as a nominal variable considering the colour scheme (black/grey; colour + black/grey; only colour).

We used conditional logistic regression for our primary analysis to align with the study protocol. In addition, we also ran unconditional logistic regression models where we broke the matching sets to optimize precision. Because we used balanced matching (case: control ratio 1:3), we accounted for the original confounding and the selection bias introduced by the matching by adjusting for sex, and for age (in 5-year intervals) with a term for residual age [25]. In the fully adjusted model, we further adjusted for household disposable income in the year before the index year (categorized according to quartiles according to the distribution among controls), marital status (married or registered partner; divorced or widowed; unmarried), educational level at the index year (primary and lower secondary; secondary; post-secondary), UV-exposure index (high; medium; low exposure), phenotypic risk index (high; medium; low risk), and smoking (current; previously; never) according to the DAG in Figure S2.

There were very few observations with missing data (1%) and we therefore restricted the analyses to observations with complete sets of covariates.

We explored the potential role of UV radiation and laser removal of tattoos as effect modifiers by introducing an interaction term between the dichotomous exposure variable and the UV-exposure index and constructing the exposure variable to distinguish tattooed individuals with respect to laser removal of tattoos, in separate models, respectively.

To clarify whether tattooed individuals had an increased risk of specific cutaneous melanoma subtypes, we performed subgroup analyses. First, we ran the analyses restricted to diagnostic codes for in situ/precursor lesions of cutaneous melanomas and invasive cutaneous melanomas, and then we restricted to melanocytic nevus with severe atypia and superficial spreading melanoma.

In sensitivity analyses, we first explored the impact of exposure duration by categorizing the exposure variable according to the number of years that had elapsed between the first tattoo and the index date (0–5; 5–10; 10–15; >15 years). Then, we performed a sensitivity analysis where we included sunburn during childhood (up to 13 years of age, many times; occasionally; never) as a covariate. Furthermore, to investigate the risk of CM in individuals with first ever diagnosis, we excluded those (*n* = 182) cases that had a previous CM diagnosis and performed analysis with only first ever diagnosis cases. To account for effects from immunosuppressive medication, we excluded individuals on such medications in another sensitivity analysis. Individuals with risk occupations associated with an increased risk for CM (firefighters and petroleum refinement workers) were excluded in a subsequent sensitivity analysis. Finally, we performed a sensitivity analysis where we included responses from deceased cases’ next-of-kin. This analysis could not be adjusted for phenotypic risk index, because next-of-kin received a reduced questionnaire that did not contain this information, and the data only allowed for an unmatched analysis.

## Results

### Study participants

**Fig. 1:**
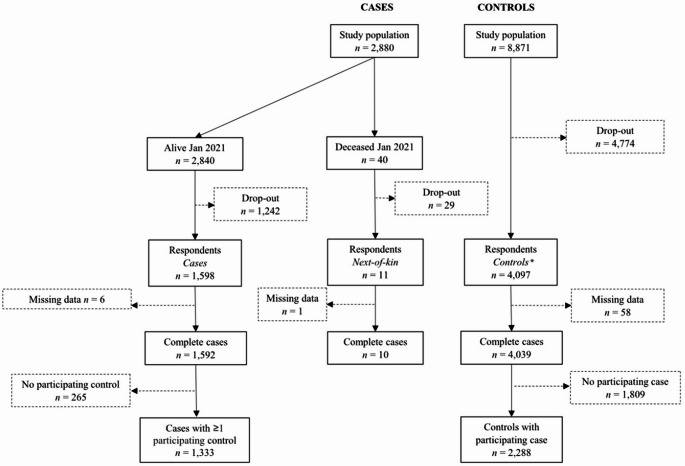
Flow chart describing the inclusion of study participants. *Number of control respondents included in crude unmatched analysis were *n* = 4018. The remaining *n* = 79 controls were from risk sets with deceased cases and therefore only included in the next-of-kin sensitivity analysis

Of the identified cases, 98% were still alive when the questionnaire was distributed (Fig. 1). The response rates among cases and controls were 56% and 46%, respectively. The drop-out analysis showed that, for both cases and controls, respondents were more often female, older, married, had higher educational attainment and income, and more often born in Sweden (Supplemental Table S1).

The median age at the index year was 51 (interquartile range = 43–56). Cases had slightly higher education level and disposable household income than controls, and they were more often married (Table 2). Overall, a higher proportion of cases were categorized as high risk according to the phenotypic risk index (Table 3). Regular recreational sun travel and use of sunbeds, as well as a higher proportion of sunburn during childhood were more frequent among cases, whereas controls were more likely to have experienced occupational sun exposure (Table 4).

**Table 2:** Characteristics of the participating cases and controls in the index year (2017)^a^

	Cases *n* (%)	Controls *n* (%)
Tattoo status		
Yes	354 (22)	815 (20)
No	1244 (78)	3203 (80)
Missing	–	–
Sex		
Male	658 (41)	1607 (40)
Female	940 (59)	2411 (60)
Age (years)		
< 29	64 (4)	161 (4)
30–39	191 (12)	472 (12)
40–49	450 (28)	1137 (28)
50–59	758 (47)	1928 (48)
60+	135 (8)	320 (8)
Missing	–	–
Educational attainment		
Primary and lower secondary	75 (5)	267 (7)
Upper secondary	318 (20)	973 (24)
Post-secondary	1203 (75)	2768 (69)
Missing	2	10
Disposable income, household (SEK)		
< 330,900	297 (19)	1003 (25)
330,900–548,800	331 (21)	1010 (25)
548,900–738,000	437 (27)	994 (25)
≥ 738,100	532 (33)	1010 (25)
Missing	1	1
Marital status		
Married/registered partnership	918 (57)	2168 (54)
Divorced/widowed	209 (13)	552 (14)
Unmarried	471 (29)	1298 (32)
Missing	–	–
Smoking		
Yes, current	112 (7)	432 (11)
Yes, previously	460 (29)	1199 (30)
No, never	1024 (64)	2376 (59)
Missing	2	11
Immunosuppressive medication		
Yes	54 (3)	90 (2)
No	1544 (97)	3928 (98)
Risk occupation		
Firefighter or Petrol refinement	11 (1)	29 (1)
No risk occupation	1587 (99)	3989 (99)

^a^Disposable income was retrieved the year before the index year

**Table 3:** Phenotypic characteristics of the cases and controls

Phenotype	Cases, *n* (%)	Controls, *n* (%)
Eye colour		
Blue	842 (53)	1796 (45)
Grey	33 (2)	100 (3)
Green	174 (11)	419 (10)
Yellow	1 (0.06)	5 (0.1)
Light brown	68 (4)	252 (6)
Dark brown	95 (6)	504 (13)
Mixed (green, blue, grey)	369 (23)	891 (22)
Different coloured eyes	8 (0.5)	27 (0.7)
Missing	8	24
Natural skin tone		
Very fair	169 (11)	229 (6)
Fair	940 (59)	2007 (51)
Medium	463 (29)	1590 (40)
Olive/light brown	10 (0.6)	103 (3)
Brown	3 (0.2)	23 (0.6)
Dark brown	0 (0)	15 (0.4)
Missing	13	51
Freckles after sun exposure		
Yes, many	260 (16)	341 (9)
Yes, a few	604 (38)	1272 (32)
No, I don’t get freckles	733 (46)	2389 (60)
Missing	1	16
Skin reaction to first sun exposure		
Burns (sometimes with blisters) and pain for at least 48 h	27 (2)	57 (1)
Burns with pain/sting that disappears within 24 h	599 (38)	1083 (27)
Initially burns, then tans	885 (56)	2382 (60)
Tans immediately	79 (5)	459 (12)
Missing	8	37
Phenotypic risk index		
High risk	630 (39)	1149 (29)
Medium risk	908 (57)	2490 (62)
Low risk	59 (4)	371 (9)
Missing	1	8

**Table 4:** Self-reported UV-exposure at the time of the survey

	Cases, *n* (%)	Controls *n* (%)
Sunburn in childhood < 13 years of age		
Yes, many times	240 (16)	271 (7)
Yes, occasionally	650 (43)	1187 (32)
No, never	622 (41)	2291 (61)
Missing	86	269
Occupational sun exposure (April-September)		
Yes	280 (18)	835 (21)
No	1217 (76)	2857 (71)
I don’t work	94 (6)	306 (8)
Missing	7	20
Weekly hours of outside work^a^		
> 20 h/week	71 (24)	247 (28)
6–20 h/week	148 (49)	382 (44)
≤ 5 h/week	80 (27)	243 (28)
Recreational sun travel > 13 years of age		
Yes, every year	445 (28)	922 (23)
Yes, occasionally	976 (62)	2409 (61)
No, never	162 (10)	648 (16)
Missing	15	39
Sunbed use > 13 years of age		
Several times a year	403 (26)	609 (15)
Occasionally	864 (54)	2232 (56)
Never	321 (20)	1144 (29)
Missing	10	33
UV-exposure index		
Ever high exposure	736 (46)	1531 (38)
Never high exposure, ever medium exposure	812 (51)	2195 (55)
Never high nor medium exposure	47 (3)	276 (7)
Missing	–	–

^a^Among those stating occupational sun exposure

The overall tattoo prevalence was 21%. Among the cases, 354 respondents (22%) were tattooed before the index year, whereas the corresponding figure among controls was 815 (20%).

The median age for first tattoos was similar between cases (24 years) and controls (25 years, Table 5). Having a combination of black and coloured ink in tattoos was most common. Taken together, the variables estimating the total area the tattooed body surface indicated somewhat smaller tattoos among cases. It was most common to have a decorative tattoo, tattooed by a professional tattoo artist in Sweden. The proportions that were tattooed by a nonprofessional or outside Europe were similar between cases and controls.

**Table 5:** Exposure characteristics among tattooed cases (*n* = 354) and controls (*n* = 815)

	Cases *n* (%)	Controls *n* (%)
Age at first tattoo (median, 25th −75th percentile)	24 (18–35)	25 (18–35)
Type of tattoo^a^		
Decorative	338 (95)	764 (94)
Cosmetic	29 (8)	76 (9)
Medical	10 (3)	15 (2)
Missing	20	41
Size		
> 5 palm	35 (10)	94 (12)
1–5 palms	126 (36)	318 (39)
< 1 palm	191 (54)	397 (49)
Missing	2	6
Colour		
Only black/grey	149 (42)	338 (42)
Both black/grey and colour	165 (47)	384 (47)
Only colour	39 (11)	90 (11)
Missing	1	3
Number of sessions		
1	143 (41)	316 (39)
2–3	123 (35)	266 (33)
4–5	35 (10)	100 (12)
6–9	26 (7)	76 (9)
>=10	24 (7)	53 (7)
Missing	3	4
Tattooist		
Professional, studio	319 (90)	712 (87)
Professional, other facility	35 (10)	93 (11)
Cosmetic tattooist, in studio or clinic	23 (7)	59 (7)
Healthcare professional, in clinic	11 (3)	12 (1)
Nonprofessional, irrespective of location	25 (7)	64 (8)
Missing	–	–
Geographical region^b^		
Sweden	314 (89)	715 (88)
Nordics, except Sweden	28 (8)	49 (6)
Rest of Europe	33 (9)	79 (10)
Asia	26 (7)	55 (7)
Oceania	5 (1)	5 (0.6)
USA	9 (3)	18 (2)
Other	6 (2)	25 (3)
Missing	–	–

^a^Multiple choice questions: percentages were calculated by the total number of tattooed cases/controls in the denominator

Among the 270 (76%) of the tattooed cases, we had information about the anatomical location of the malignancy. In 30%, the tattoo was placed on the same anatomical location as the malignancy, although the correlation was low (Spearman’s rho = 0.007, *p*-value = 0.91).

### Main analyses: tattoos and cutaneous melanoma

We found a 29% increased risk (IRR = 1.29, 95% CI: 1.07–1.56) of overall cutaneous melanoma among tattooed individuals in the matched adjusted analysis (Table 6). In the unmatched analysis, the risk was slightly attenuated (IRR = 1.24, 95% CI: 1.06–1.44). We did not find that larger tattoos resulted in a higher risk (Table 6). We found that individuals with tattoos that were both black/grey and coloured (IRR = 1.38, 95% CI: 1.08–1.77) had a higher risk compared to nontattooed individuals, and individuals with coloured tattoos also seemed to be at a similar risk, although the point estimate was associated with a wide confidence interval because of small numbers. We found a consistent risk increase over all exposure duration intervals, except 5–10 years where the estimate was null (Table 6). Both shorter exposure duration 0–5 years and a longer exposure duration (10–15 years) were associated with increased risks, IRR = 1.60 (95% CI: 0.99–2.58) and IRR = 1.67 (95% CI: 1.09–2.56), respectively. Individuals with over 15 years of tattoo exposure had a slightly lower risk increase (IRR = 1.24, 95% CI: 0.98–1.57). In contrast, the unmatched analysis showed a consistent risk increase over all exposure durations. We did not find evidence of effect modification by UV exposure (P[interaction] = 0.58. Table S2). Adding childhood sunburn to the model increased the effect estimate to 1.31 (95% CI 1.07–1.61) (Table 6). Very few participants had removed tattoos using laser, and their effect estimates were below 1 and associated with a very wide confidence interval (Table 6).

**Fig. 2:**
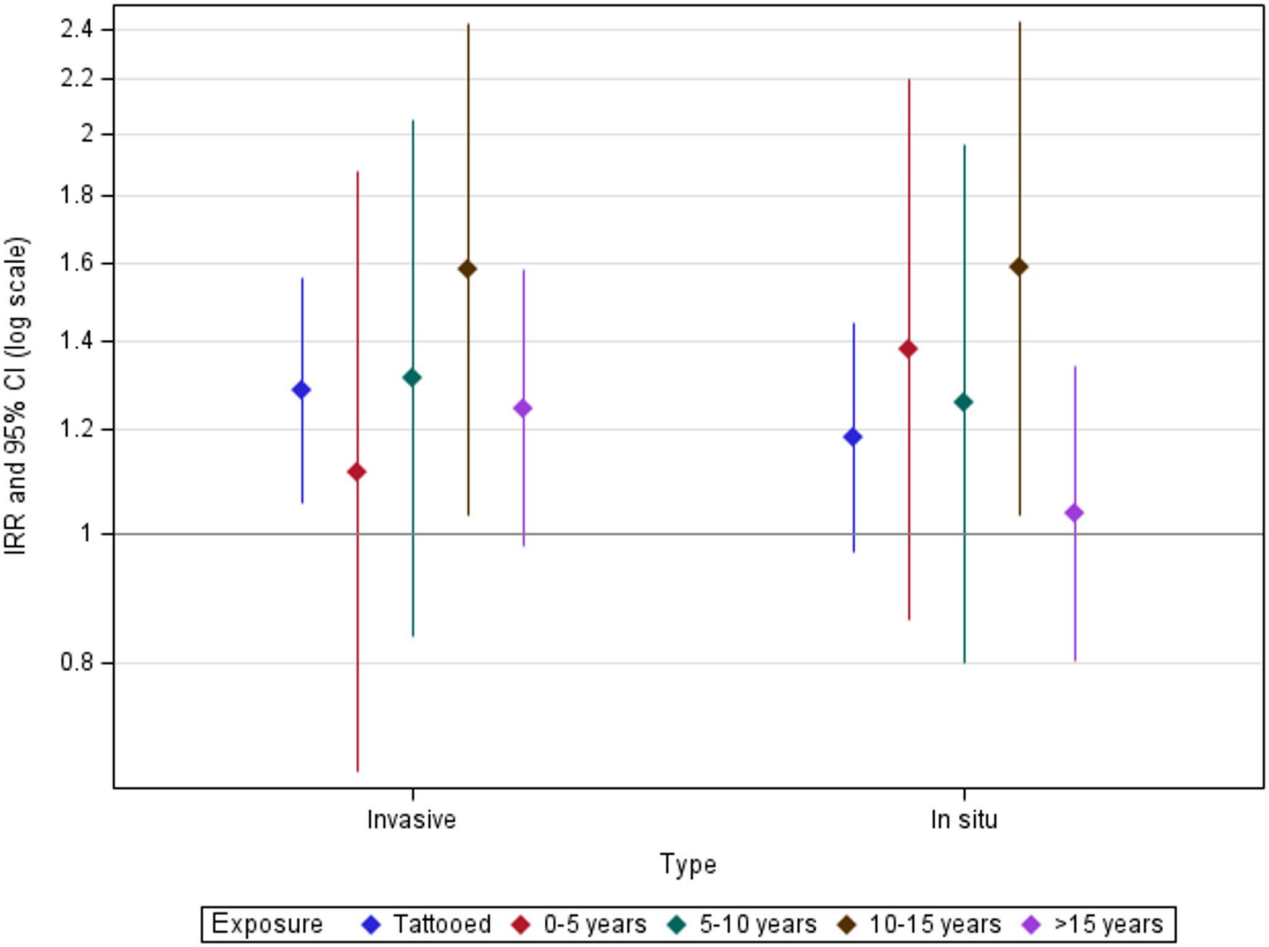
Adjusted incidence rate ratios (IRR) of cutaneous melanoma subgroups relative to non-tattooed participants. Tattoo exposure was modelled as exposure status (tattooed vs. nontattooed), and exposure duration (0-5[red], 5-10[green], 10-15[brown], >15 years[purple] vs. non-tattooed)

We found increased risks for both subgroups of cutaneous melanoma; invasive cutaneous melanomas (IRR = 1.25, 95% CI = 0.95–1.64) and in situ cutaneous melanomas (IRR = 1.33, 95% CI = 1.02–1.75) (Fig. 2, Table S3). When analysing the risk for the single largest type in each subgroup, we found even higher risk estimates; superficial spreading melanoma (IRR = 1.40, 95% CI = 1.03–1.90) and melanocytic nevus with severe atypia (IRR = 1.39, 95% CI = 1.05–1.85). An exposure duration of 10 to 15 years was associated with the highest point estimates irrespective of cutaneous melanoma subtype.

When excluding individuals who had a previous CM diagnosis (Table S4), we found increased risks for first CM diagnosis cases (IRR = 1.23, 95% CI = 1.01–1.51), however lower than the main results. When excluding individuals with immunosuppressive medications, we found that the risk increased further (IRR = 1.32, 95% CI = 1.10–1.62). When excluding individuals with risk occupations risk estimates did not deviate from the main result.

Including the responses from the 11 participating next-of-kin did not affect the results (Table S5).

**Table 6:** Incidence rate ratios (IRR) of cutaneous melanoma in tattooed individuals relative to non-tattooed individuals

	Matched analysis						Unmatched analysis					
	Basic adjustment^a^			Full adjustment^b^			Basic adjustment^c^			Full adjustment^d^		
	Cases (*n*)	Controls (*n*)	IRR^e^ (95% CI)	Cases (*n*)	Controls (*n*)	IRR (95% CI)	Cases (*n*)	Controls (*n*)	IRR (95% CI)	Cases (*n*)	Controls (*n*)	IRR (95% CI)
Tattoo status (main analysis)	1333	2271		1328	2243		1598	4018		1592	3979	
Tattooed	285	437	1.13 (0.95–1.34)	284	432	1.29 (1.07–1.56)	354	815	1.13 (0.98–1.30)	353	809	1.24 (1.06–1.44)
Non-tattooed	1048	1834	1.00	1044	1811	1.00	1244	3203	1.00	1239	3170	1.00
Tattooed body surface	1331	2268		1327	2240		1596	4012		1591	3973	
Tattooed body surface < 1 palm	159	216	1.31 (1.05–1.68)	159	213	1.47 (1.16–1.87)	191	397	1.25 (1.04–1.51)	191	394	1.33 (1.10–1.61)
Tattooed body surface > 1 palms	124	218	0.95 (0.75–1.22)	124	216	1.12 (0.86–1.45)	161	412	1.01 (0.83–1.23)	161	409	1.15 (0.93–1.42)
Non-tattooed	1048	1834	1.00	1044	1811	1.00	1244	3203	1.00	1239	3170	1.00
Ordinal exposure variable	1330	2268		1326	2240		1595	4014		1590	3975	
1 tattoo sessions	121	178	1.19 (0.93–1.53)	121	173	1.32 (1.01–1.73)	143	316	1.17 (0.95–1.45)	143	311	1.28 (1.03–1.59)
2–3 tattoo sessions	96	142	1.17 (0.89–1.55)	96	142	1.37 (1.02–1.84)	123	266	1.20 (0.96–1.50)	123	266	1.29 (1.03–1.63)
>4 tattoo sessions	65	114	0.96 (0.69–1.34)	65	114	1.14 (0.81–1.61)	85	229	0.96 (0.74–1.25)	85	228	1.10 (0.83–1.44)
Non-tattooed	1048	1834	1.00	1044	1811	1.00	1244	3203	1.00	1239	3170	1.00
Colour	1332	2269		1327	2241		1597	4015		1591	3976	
Black/grey	111	180	1.04 (0.80–1.34)	110	178	1.17 (0.88–1.54)	149	338	1.15 (0.94–1.42)	148	335	1.23 (0.99–1.53)
Black/grey + colour	142	209	1.17 (0.93–1.47)	142	209	1.38 (1.08–1.77)	165	384	1.11 (0.92–1.35)	165	384	1.27 (1.04–1.56)
Colour only	31	46	1.31 (0.82–2.11)	31	43	1.40 (0.85–2.31)	39	90	1.11 (0.76–1.63)	39	87	1.17 (0.79–1.73)
Non-tattooed	1048	1834	1.00	1044	1811	1.00	1244	3203	1.00	1239	3170	1.00
Exposure duration	1333	2271		1328	2243		1598	4018		1592	3979	
0–5 years	38	48	1.44 (0.91–2.28)	38	47	1.60 (0.99–2.58)	45	98	1.19 (0.82–1.72)	45	97	1.25 (0.85–1.83)
5–10 years	37	70	0.92 (0.61–1.40)	36	70	0.98 (0.63–1.53)	55	125	1.17 (0.84–1.64)	54	125	1.27 (0.90–1.79)
10–15 years	49	55	1.50 (1.00–2.25.00.25	49	55	1.67 (1.09–2.56)	62	110	1.50 (1.08–2.07)	62	110	1.60 (1.15–2.24)
Over 15 years	161	264	1.06 (0.85–1.31)	161	260	1.24 (0.98–1.57)	192	482	1.03 (0.86–1.23)	192	477	1.15 (0.95–1.39)
Non-tattooed	1048	1834	1.00	1044	1811	1.00	1244	3203	1.00	1239	3170	1.00
Childhood sunburn^f^	1333	2271		1252	2098		1598	4018		1507	3722	
Tattooed	285	437	1.13 (0.95–1.34)	270	403	1.31 (1.07–1.61)	354	815	1.13 (0.98–1.30)	334	748	1.23 (1.05–1.44)
Non-tattooed	1048	1834	1.00	982	1695	1.00	1244	3203	1.00	1173	2974	1.00
Laser removal	1331	2268		1327	2240		1596	4012		1591	3974	
Tattooed, laser removal	5	16	0.44 (0.16–1.24)	5	16	0.64 (0.22–1.82)	7	25	0.73 (0.31–1.69)	7	25	0.85 (0.36–2.01)
Tattooed, no laser removal	278	418	1.16 (0.97–1.38)	278	413	1.32 (1.09–1.60)	345	784	1.14 (0.99–1.32)	345	779	1.25 (1.08–1.46)
Non-tattooed	1048	1834	1.00	1044	1811	1.00	1244	3203	1.00	1239	3170	1.00

^a^Estimates obtained from conditional logistic regression adjusted for sex and age

^b^Estimates obtained from conditional logistic regression adjusted for age, sex, educational attainment, household disposable income, marital status, phenotypic risk index, UV-exposure index, and smoking

^c^Estimates obtained from unconditional logistic regression adjusted for sex and age

^d^Estimates obtained from unconditional logistic regression adjusted for age, sex, educational attainment, household disposable income, marital status, phenotypic risk index, UV-exposure index, and smoking

^e^Incidence rate ratio

^f^In addition to ^b^ and ^d^, also adjusted for childhood sunburn

## Discussion

The results suggested that tattooed individuals had a 29% increased risk of cutaneous melanoma compared to nontattooed individuals. When only cases with a first diagnosis were included, tattooed individuals had a 23% increased risk for cutaneous melanoma. According to various studies and meta-analyses, the risk associated with UV radiation—the most well-established risk factor of cutaneous melanoma—is estimated to be comparable to up to five times higher [26–30] than the risk observed in our study. If tattoo exposure is an additional risk factor for cutaneous melanoma, it may exacerbate the risk imposed by UV exposure when tattooed skin is exposed to the sun.

We did not find that highly UV-exposed tattooed individuals had a higher risk of cutaneous melanoma than tattooed individuals with lower UV exposure. Thus, our data suggests that UV-accelerated pigment breakdown does not increase the risk of cutaneous melanoma beyond the risk associated with tattoo exposure per se, but further studies in more UV-intense regions of the world is warranted.

In 2012, a literature review of case reports [17] concluded that the link between tattoos and skin cancer was likely coincidental. The authors reached this conclusion by comparing the number of published case reports of cutaneous malignancies in tattoos with the size of the estimated global tattooed population. However, case studies suffer from inherent publication bias due to selective reporting and cannot be used to evaluate associations between exposures and outcomes. Thus, our results provide the first insight into a possible association between tattoos and skin cancer using an adequate methodological approach.

We did not see a higher risk increase for those with larger areas of tattooed skin, rather their IRRs were lower than those of individuals with smaller tattoos. It is possible there was misclassification in the ordinal exposure variable because tattooed individuals tend to overestimate their tattooed body area [24]. It could also be caused by variation in the amount of ink needed for tattooing [31]. In the matched analysis, coloured tattoos alone or in combination with black or grey in the tattoo seemed to be associated with a slightly higher risk of cutaneous melanoma. However, statistical power for the group with only coloured tattoos was an issue, and further investigation is warranted to clarify if there is a difference in cancer risk depending on the ink colour.

Depending on the mechanism of carcinogenicity, chemicals can act as tumour initiators or tumour promotors [32], either initiating the cancerous development of a cell – which would indicate a longer latency period between exposure and diagnosis – or promoting or progressing its development resulting in a shorter latency period. The biological mechanism underlying cancer development should inform the analytical choices regarding latency times but given the complex mixture of chemicals in tattoo ink, this was not possible. Instead, we chose to adjust for exposure duration. We found that those with an exposure duration of 10–15 years were at highest risk of developing cutaneous melanoma. The same time span is generally implicated in UV-induced cutaneous melanoma. We also observed high risk estimates in individuals with short exposure duration (up to five years), but the confidence interval was wide. Mechanistically, the results suggest that tattoo exposure could be associated with tumour initiation, and possibly with tumour promotion, but future research is needed to confirm this for example by longitudinal studies following tattooed individuals.

In our previous study on the association of cutaneous squamous cell carcinoma (CSCC) and tattoos, using the same study design as in the present study, we did not find an increased risk for tattooed individuals [33]. Nielsen et al. [34] found an increased risk for lymphoma for tattooed individuals, also using the same study design as in the present study, and Clemmensen et al. [18] found evidence for an increased risk for tattooed individuals for lymphoma and skin cancers in a Danish twin study. There now are three epidemiological studies suggesting an association between tattoos and cancer – lymphoma, and cutaneous melanoma. These are both cancers with a strong immunologic component, while SCC is not. Cutaneous melanoma is a highly immunogenic cancer with high infiltrates of immune cells, and sophisticated evasion tactics, that respond well to immunotherapy [35–38]. Squamous cell carcinoma, while still influenced by the immune system, is less immunogenic, with less immune infiltrates and has less complex immune evasion tactics, that responds less well to immunotherapy [39, 40]. We propose that carcinogenic mechanisms may stem from immunotoxicity of tattoo ink. Studies using reconstructed human skin show that tattoo ink can present immunotoxic properties such as cytotoxicity and act as sensitizers [41, 42], which might provide a pathobiological mechanism to explain the epidemiologic results, and why we see an increased risk for CM and not for cSCC. Our argument is further supported by the stronger association observed when we exclude participants on immunosuppressive medications. This exclusion results in an even stronger association and a 32% increase in risk.

In the subgroup analysis, we found that the risk associated with tattoo exposure seemed to be increased for both main groups: invasive and in-situ cutaneous melanomas, attributable to both superficial spreading melanoma and melanocytic nevus with severe atypia. Tattooed individuals had an approximate risk increase of 40% compared to nontattooed individuals for both subtypes. Superficially spreading melanomas constitute an invasive cancer, which often shows histopathological remnants of melanocytic nevi in part of the lesion [43]. Therefore, it may be advisable to take extra precautions when clinically evaluating nevi and the skin in tattooed individuals. After excluding cases with previous diagnosis of CM, the risk increase was lower (23%) than for the entire group (29%). Cases with multiple diagnosis of CM is likely a group with many life-style related and genetic risk factors, which may affect the risk estimate, as well as decreased power when excluding 182 individuals and their matched controls, which could explain the slightly lower risk increase of 23%.

Tattoo-related factors, other than the pigments as such, that could theoretically play a role in the carcinogenesis are the trauma induced by puncturing the skin, the scarring process, and exposure to solar irradiation when tattoos are not covered with clothing [17]. Biomolecular changes indicative of chronic inflammation have been described in skin adjacent to tattoo pigments [44], and the role of inflammation in the development of cancer is well established [45]. Only 30% of the tattoos were located on the same anatomical site as the malignancy. This may seem illogical, but studies of firefighters [46] and petroleum workers [47, 48] have demonstrated increased risks of cutaneous melanomas despite the fact that their exposure to chemicals (including, e.g., PAH) usually is more systemic, through uptake via lungs and skin [49, 50], and not necessarily localized to the anatomical location of the malignancy. Another study of Danish twins found the same association, although for both melanoma and non-melanoma skin cancers [18].

It is not clear for how long exposure to soluble ink components can be expected systemically, but systemic exposure may not be necessary. Instead, the possible effects that components of tattoo ink could exert on the immune system locally (i.e. at the site of the tattoo, and in the proximate lymph nodes) may become systemic. However, the toxicokinetics of tattoo ink exposure is an understudied area, and a proper risk assessment can be made once more is known about the kinetics and dynamics of tattoo ink in the body.

### Strengths and limitations

Our study is the first epidemiological study to investigate the association between tattoos and cutaneous melanoma using a large population-based sample with access to information about important confounders. We consider the exposure variable – having a tattoo – to be non-sensitive to recall bias owing to the invasive nature of tattoos. Importantly, we found that common risk factors for cutaneous melanoma, such as high UV exposure and light skin, were more common among cases than controls, which suggests that our results are valid. Given the response rate of 50% in the present study, selection bias could have been introduced if the likelihood of responding was related to both the exposure and the outcome. However, to decrease the risk of conditioning participation on exposure or outcome status, the questionnaire asked about several lifestyle-related factors and informed participants that the purpose of the study was to investigate the association between lifestyle-related factors and cancer as well as other diseases.

A limitation may be that responders and non-responders differed with respect to SES. Because we assume that UV exposure is dependent on SES, selection bias with respect to SES could have affected the estimates, and particularly the lack of interaction we found for tattoos and UV-exposure. The results may not be generalizable to older age groups, given the different presentation, clinical course and prognosis of cutaneous melanoma [51]. Caution should be taken in generalizing our results to a setting with higher UV exposure, because we could only investigate the potential effect modification in a setting with relatively low UV exposure.

Childhood sun exposure is a known risk factor for CM [27, 52], and in the context of our investigation on CM risk for tattooed individuals, it is likely an important precision variable. The item reflecting childhood sun exposure relevant to our investigation was childhood sunburn. Our risk and sun indexes did not include childhood sunburn, because we wanted them to reflect the participants’ risk behaviour in adult life. We also decided not to include childhood sunburn in the main analysis, because of the higher missingness in the variable. However, after adding childhood sunburn to a sensitivity analysis, we found that this increased the risk for tattooed individuals further, and we may therefore underestimate the CM risk for tattooed individuals in our main analysis.

Our data included mainly melanocytic nevi with severe atypia and superficial spreading melanoma. Therefore, we cannot draw conclusions as to the association between tattoo exposure and the less common types of cutaneous melanomas, because of limited statistical power.

## Conclusions

The results suggested that tattoos may be a risk factor for cutaneous melanoma. The results need to be verified in additional epidemiologic studies before causality can be inferred.

## Supplementary Information

Below is the link to the electronic supplementary material.

Supplementary Material 1

## Data Availability

The data underlying this article cannot be shared publicly due to the privacy of the. participants. Register data are obtained from third parties and are not publicly available.
