# Supplementary material for: Does tattoo exposure increase the risk of cutaneous melanoma? A population-based case-control study
Source: Eur J Epidemiol. 2025 Nov 24;40(12):1441–53. doi: 10.1007/s10654-025-01326-6 (PMC12756314; doi:10.1007/s10654-025-01326-6)
Supplement: Supplementary file 1 — Supplementary Material 1 [file 10654_2025_1326_MOESM1_ESM.docx]

# Supplemental material to ”Does tattoo exposure increase the risk of cutaneous melanoma? A population-based case-control study”

Emelie Rietz Liljedahl^1, PhD^, Kari Nielsen^3,4, PhD^, Malin Engfeldt^1,2, PhD^, Anna Jöud^1,5, PhD^, and Christel Nielsen^1,6, PhD^

^1^Division of Occupational and Environmental Medicine, Department of Laboratory Medicine, Lund University, Lund, Sweden

^2^Department of Occupational and Environmental Medicine, Region Skåne, Lund, Sweden

^3^Department of Dermatology, Skåne University Hospital, Lund, Sweden

^4^Dermatology, Department of Clinical Sciences, Lund University, Lund, Sweden

^5^ Department of Clinical Sciences, Lund University, Lund, Sweden

^6^Clinical Pharmacology, Pharmacy and Environmental Medicine, Institute of Public Health, University of Southern Denmark, Odense, Denmark


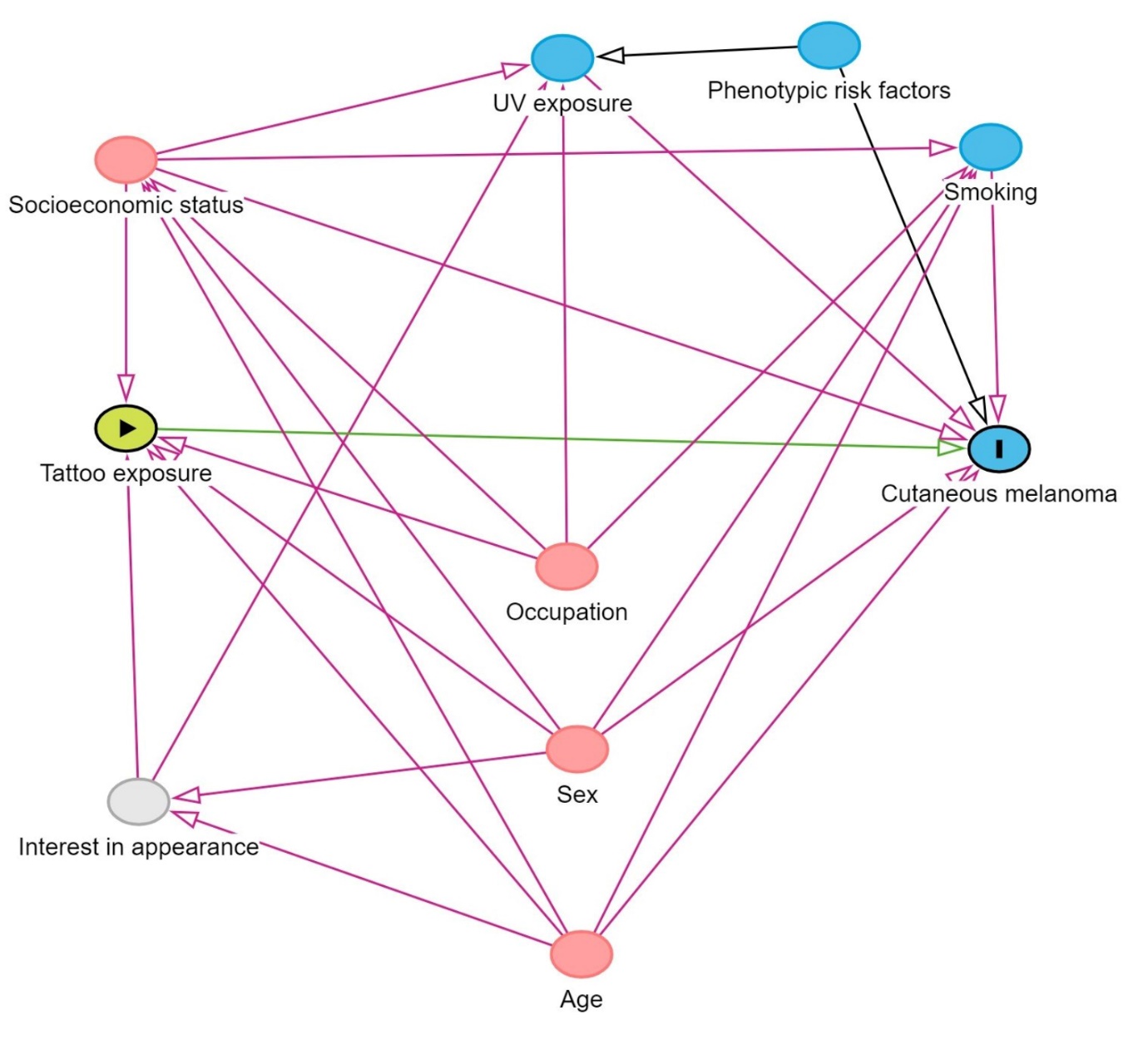


Figure S1. The directed acyclic graph visualizing the underlying associations between tattoos and cutaneous melanoma, and potential measurable and unmeasurable confounders related to tattoo exposure and/or melanoma.


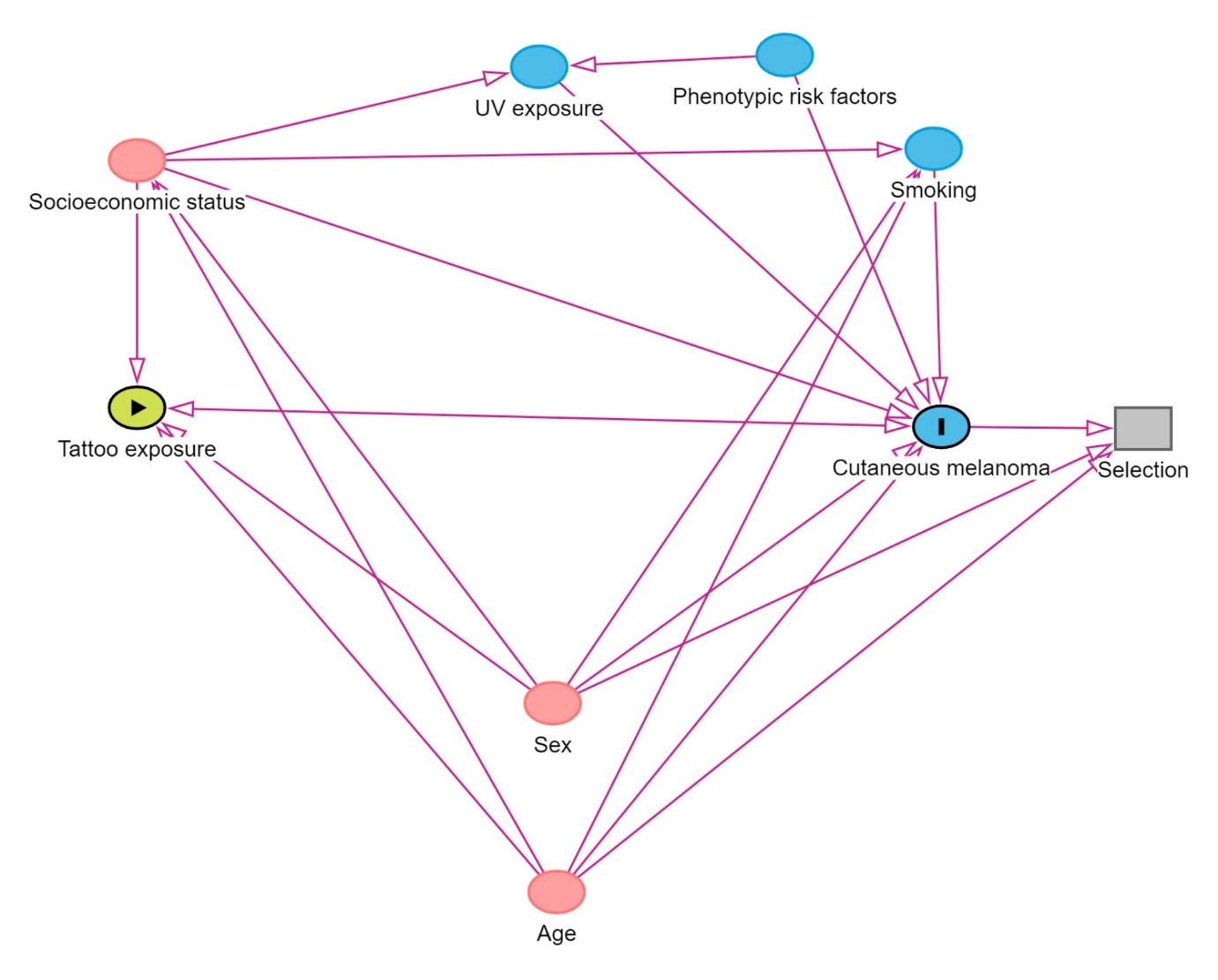


Figure S2.The directed acyclic graph used to inform the statistical analyses.

Table S1. Descriptive drop-out analysis of cases and controls, displayed as n (%) at the time of the survey.

|  | Cases^a^ |  | Controls |  |
| --- | --- | --- | --- | --- |
|  | Respondents | Non-respondents | Respondents | Non-respondents |
| Sex |  |  |  |  |
| Male | 658 (53) | 586 (47) | 1640 (43) | 2172 (57) |
| Female | 940 (57) | 710 (43) | 2458 (49) | 2602 (51) |
| Age |  |  |  |  |
| 20-29 | 28 (42) | 38 (58) | 62 (30) | 142 (70) |
| 30-39 | 135 (40) | 199 (60) | 349 (34) | 676 (66) |
| 40-49 | 333 (49) | 342 (51) | 830 (40) | 1220 (60) |
| 50-59 | 658 (57) | 506 (43) | 1745 (49) | 1806 (51) |
| 60-69 | 444 (68) | 211 (32) | 1112 (54) | 930 (46) |
| Educational attainment |  |  |  |  |
| Primary and lower secondary | 75 (41) | 110 (59) | 273 (28) | 694 (72) |
| Upper secondary | 618 (50) | 628 (50) | 1738 (42) | 2361 (58) |
| Post-secondary | 903 (62) | 553 (38) | 2081 (56) | 1631 (44) |
| Missing | 2 (33) | 4 (67) | 6 (6) | 88 (95) |
| Country of birth |  |  |  |  |
| Sweden | 1529 (56) | 1209 (44) | 3545 (51) | 3390 (49) |
| Other | 69 (44) | 87 (56) | 553 (29) | 1384 (72) |
| Marital status |  |  |  |  |
| Married | 945 (62) | 582 (38) | 2270 (52) | 2101 (48) |
| Unmarried | 431 (46) | 509 (54) | 1231 (41) | 1769 (59) |
| Divorced | 209 (52) | 190 (48) | 551 (40) | 841 (60) |
| Widowed | 13 (46) | 15 (54) | 46 (42) | 63 (58) |
| Disposable income (SEK^2^) |  |  |  |  |
| None (0) | 13 (36) | 23 (64) | 82 (22) | 287 (78) |
| 1 – 124,999 | 67 (44) | 84 (56) | 198 (29) | 477 (71) |
| 125,000 – 199,999 | 72 (45) | 88 (55) | 241 (36) | 427 (64) |
| 200,000 – 279,999 | 135 (47) | 150 (53) | 423 (41) | 607 (59) |
| 280,000 – 369,999 | 324 (52) | 296 (48) | 905 (45) | 1094 (55) |
| ≥370,000 | 987 (60) | 655 (40) | 2249 (54) | 1882 (46) |

^a^Not including deceased cases.

| Matched analysis^a^ | | | | Unmatched analysis^b^ | | |
| --- | --- | --- | --- | --- | --- | --- |
| Interaction | Cases (*n*) | Controls (*n*) | IRR (95% CI) | Cases (*n*) | Controls (*n*) | IRR (95% CI) |
| High UV, tattooed | 159 | 202 | 1.94 (1.5-2.5) | 196 | 380 | 1.79 (1.46-2.20) |
| High UV, non-tattooed | 459 | 646 | 1.41 (1.12-1.67) | 538 | 1144 | 1.40 (1.22-1.61) |
| Low-medium UV, tattooed | 125 | 230 | 1.24 (0.95-1.62) | 157 | 429 | 1.23 (1.00-1.52) |
| Low-medium UV, non-tattooed | 585 | 1165 | 1.00 | 701 | 2026 | 1.00 |
| ^a^Estimates obtained from conditional logistic regression adjusted for age, sex, educational attainment, household disposable income, marital status, phenotypic risk index, UV-exposure index, and smoking. ^b^Estimates obtained from unconditional logistic regression adjusted for age, sex, educational attainment, household disposable income, marital status, phenotypic risk index, UV-exposure index, and smoking. | | | | | | |

Table S2. Adjusted incidence rate ratios of cutaneous melanoma in tattooed and non-tattooed individuals with different degrees of sun exposure, expressed in relation to non-tattooed individuals with low UV exposure from interaction analysis.

*Table S3. Exploratory subgroup analyses of the association between a) tattoo exposure status, and b) tattoo exposure duration, and melanoma subtypes.*

|  |  | Matched analysis | | | Unmatched analysis | | |  | Unmatched analysis | | |
| --- | --- | --- | --- | --- | --- | --- | --- | --- | --- | --- | --- |
|  |  | Full adjustment^a^ | | | Full adjustment^b^ | | |  | Full adjustment^b^ | | |
|  | Exposure status | Cases (*n*) | Controls (*n*) | IRR^c^(95 % CI) | Cases (*n*) | Controls (*n*) | IRR(95 % CI) | Exposure duration | Cases (*n*) | Controls (*n*) | IRR (95 % CI) |
| *Invasive melanoma^d^* | Tattooed | 145 | 432 | 1.25 (0.95-1.64) | 182 | 809 | 1.29 (1.06-1.56) | 0-5 | 19 | 97 | 1.12 (0.66-1.88) |
|  |  |  |  |  |  |  |  | 5-10 | 27 | 125 | 1.31 (0.84-2.05) |
|  |  |  |  |  |  |  |  | 10-15 | 31 | 110 | 1.59 (1.04-2.43) |
|  |  |  |  |  |  |  |  | >15 | 105 | 477 | 1.25 (0.98.1.58) |
|  | Non-tattooed | 521 | 1811 | 1.00 | 617 | 3170 | 1.00 | Non-tattooed | 617 | 3170 | 1.00 |
| *Superficial spreading melanoma* | Tattooed | 122 | 432 | 1.40 (1.03-1.90) | 149 | 809 | 1.25 (1.02-1.53) | 0-5 | 16 | 97 | 1.20 (0.69-2.08) |
|  |  |  |  |  |  |  |  | 5-10 | 20 | 125 | 1.26 (0.77-2.08) |
|  |  |  |  |  |  |  |  | 10-15 | 25 | 110 | 1.66 (1.05-2.62) |
|  |  |  |  |  |  |  |  | >15 | 88 | 477 | 1.36 (1.05-1.76) |
|  | Non-tattooed | 405 | 1811 | 1.00 | 476 | 3170 | 1.00 | Non-tattooed | 476 | 3170 | 1.00 |
| *In situ melanoma^e^* | Tattooed | 139 | 432 | 1.33 (1.02-1.75) | 171 | 809 | 1.18 (0.97-1.45) | 0-5 | 26 | 97 | 1.38 (0.86-2.21) |
|  |  |  |  |  |  |  |  | 5-10 | 27 | 125 | 1.26 (0.80-1.97) |
|  |  |  |  |  |  |  |  | 10-15 | 31 | 110 | 1.59 (1.04-2.43) |
|  |  |  |  |  |  |  |  | >15 | 87 | 477 | 1.04 (0.80-1.34) |
|  | Non-tattooed | 523 | 1811 | 1.00 | 622 | 3170 | 1.00 | Non-tattooed | 622 | 3170 | 1.00 |
| *Melanocytic nevus* | Tattooed | 126 | 432 | 1.39 (1.05-1.85) | 156 | 809 | 1.18 (0.96-1.45) | 0-5 | 25 | 97 | 1.47 (0.91-2.36) |
|  |  |  |  |  |  |  |  | 5-10 | 23 | 125 | 1.15 (0.71-1.19) |
|  |  |  |  |  |  |  |  | 10-15 | 28 | 110 | 1.51 (0.97-2.35) |
|  |  |  |  |  |  |  |  | >15 | 80 | 477 | 1.04 (0.80-1.36) |
|  | Non-tattooed | 469 | 1811 | 1.00 | 559 | 3170 | 1.00 | Non-tattooed | 559 | 3170 | 1.00 |
| ^a^Estimates obtained from conditional logistic regression adjusted for age, sex, educational attainment, household disposable income, marital status, phenotypic risk index, UV-exposure index, and smoking.^b^Estimates obtained from unconditional logistic regression adjusted for age, sex, educational attainment, household disposable income, marital status, phenotypic risk index, UV-exposure index, and smoking.Exposure duration was not analyzed with conditional logistic regression due to small sample size. ^c^Incidence rate ratio. | | | | | | | | | | | |
| ^d^Includes nevoid melanoma and melanoma not otherwise specified, nodular melanoma, lentigo maligna melanoma, superficial spreading melanoma, acral lentiginous melanoma, and mixed epithelioid, and spindle cell melanoma.^e^Includes melanocytic nevus with severe atypia, lentigo maligna *in situ*, acral lentiginous melanoma *in situ,* and atypical Spitz’s nevus. | | | | | | | | | | | |

Table S4. Incidence rate ratios (IRR) of cutaneous melanoma in tattooed individuals relative to non-tattooed individuals.

|  | Matched analysis | | | | | | Unmatched analysis | | | | | |
| --- | --- | --- | --- | --- | --- | --- | --- | --- | --- | --- | --- | --- |
|  | Basic adjustment^a^ | | | Full adjustment^b^ | | | Basic adjustment^c^ | | | Full adjustment^d^ | | |
|  | Cases (*n*) | Controls (*n*) | IRR^e^ (95 % CI) | Cases (*n*) | Controls (*n*) | IRR (95 % CI) | Cases (*n*) | Controls (*n*) | IRR (95 % CI) | Cases (*n*) | Controls (*n*) | IRR (95 % CI) |
|  |  |  |  |  |  |  |  |  |  |  |  |  |
| *First time melanoma* | 1173 | 1982 |  | 1168 | 1955 |  | 1416 | 4018 |  | 1410 | 3979 |  |
| Tattooed | 249 | 390 | 1.07 (0.89-1.29) | 248 | 386 | 1.23 (1.01-1.51) | 314 | 815 | 1.11 (0.96-1.29) | 313 | 809 | 1.21 (1.03-1.41) |
| Non-tattooed | 924 | 1592 |  | 920 | 1569 |  | 1102 | 3203 |  | 1097 | 3170 |  |
| *Immunosuppressive meds excl.* | 1285 | 2217 |  | 1280 | 2190 |  | 1544 | 3928 |  | 1538 | 3890 |  |
| Tattooed | 279 | 424 | 1.17 (0.98-1.40) | 278 | 419 | 1.32 (1.10-1.62) | 346 | 796 | 1.15 (0.99-1.33) | 345 | 790 | 1.25 (1.07-1.46) |
| Non-tattooed | 1006 | 1793 | 1.00 | 1002 | 1771 | 1.00 | 1198 | 3132 | 1.00 | 1193 | 3100 | 1.00 |
| *Risk occupation excl* | 1324 | 2261 |  | 1319 | 2234 |  | 1587 | 3989 |  | 1581 | 3951 |  |
| Tattooed | 283 | 434 | 1.12 (0.94-1.33) | 282 | 1037 | 1.29 (1.06-1.55) | 352 | 807 | 1.13 (0.98-1.31) | 351 | 801 | 1.24 (1.07-1.45) |
| Non-tattooed | 1041 | 1827 | 1.00 | 429 | 1805 | 1.00 | 1235 | 3182 | 1.00 | 1230 | 3150 | 1.00 |
| ^a^Estimates obtained from conditional logistic regression adjusted for sex and age. ^b^Estimates obtained from conditional logistic regression adjusted for age, sex, educational attainment, household disposable income, marital status, phenotypic risk index, UV-exposure index, and smoking. ^c^Estimates obtained from unconditional logistic regression adjusted for sex and age. ^d^Estimates obtained from unconditional logistic regression adjusted for age, sex, educational attainment, household disposable income, marital status, phenotypic risk index, UV-exposure index, and smoking. ^e^Incidence rate ratio. | | | | | | | | | | | | |

|  | Basic adjustment^a^ | | | Full adjustment^b^ | | |
| --- | --- | --- | --- | --- | --- | --- |
|  | Cases (*n*) | Controls (*n*) | IRR^c^ (95 % CI) | Cases (*n*) | Controls (*n*) | IRR (95 % CI) |
|  |  |  |  |  |  |  |
| Tattoo status | 1609 | 4097 |  | 1602 | 4044 |  |
| Tattooed | 354 | 826 | 1.12 (0.97-1.29) | 353 | 821 | 1.20 (1.03-1.40) |
| Non-tattooed | 1255 | 3271 | 1.00 | 1249 | 3223 | 1.00 |
| All estimates were obtained from unconditional logistic regression due to small groups of next-of-kin. | | | | | | |
| ^a^Estimates obtained from unconditional logistic regression adjusted for sex and age. ^b^Estimates obtained from unconditional logistic regression adjusted for age, sex, educational attainment, household disposable income, marital status, UV-exposure index, and smoking. ^c^Incidence rate ratio. | | | | | | |

Table S5. Incidence rate ratios (IRR) of cutaneous melanoma in tattooed individuals relative to non-tattooed individuals, including deceased cases’ next-of-kin (n=11).
